# The training and development process during childhood and adolescence of a multiple Ballon d'Or-nominated soccer player

**DOI:** 10.3389/fspor.2025.1710194

**Published:** 2025-12-11

**Authors:** Espen Tønnessen, Silvana Bucher Sandbakk, Sigmund Apold-Aasen, Øyvind Sandbakk, Thomas A. Haugen

**Affiliations:** 1 School of Health Sciences, Kristiania University of Applied Sciences, Oslo, Norway; 2 Department of Sport Science and Physical Education, University of Agder, Kristiansand, Norway; 3 School of Sport Science, UiT The Artic University of Norway, Tromsø, Norway

**Keywords:** team sport athletes, talent development environment, training recommendations, coaching strategies, longitudinal study

## Abstract

**Introduction:**

Information regarding the training and development of youth talents who successfully progress to professional soccer remains both limited and contradictory. The aim of this study was to retrospectively investigate the training and development process of a world-class soccer player during childhood and adolescence.

**Methods:**

An intrinsic case study design was employed to capture the quantitative and qualitative aspects of the training and development process. A four-step data collection procedure was used, along with pragmatic analyses of (1) training history based on logs and plans, (2) in-depth semi-structured interviews with the player's father, who also served as head coach throughout the analyzed period, (3) follow-up interviews to clarify, expand upon, or validate findings from steps 1 and 2, and (4) systematic quality assurance through triangulation and negotiation among researchers and key informants, including the player.

**Results:**

During childhood and adolescence, the player engaged in approximately 1,000–1,300 h of soccer training annually. Unorganized training constituted most of this time, though its proportion gradually declined as the volume of organized training increased. The unorganized training conducted with his father was guided by a clear philosophy: Basic technical skills were learned through isolated and targeted drills in form of ball control, passing, receiving, feints, visual exploration and scanning. These foundational skills were reinforced and refined in game-realistic settings during unorganized play with friends on the local turf, and ultimately, through match play. As the player progressed, increasingly complex tasks were introduced. He received personalized challenges that matched his current skill level and supported further development. Key developmental factors contributing to his success included a multidimensional motor talent, an exceptional passion for soccer and a strong willingness to train, a learning-oriented mindset, training with 1–3-year older peers, a supporting and knowledgeable father, and year-round access to high-quality training facilities.

**Conclusion:**

The novel insights reported here may serve as a basis for reflection when players and coaches consider how they should optimize the long-term development and performance. However, the unusually high training volumes described here should not be interpreted as general recommendations for average youth players or non-elite settings.

## 1 Introduction

Soccer is the world's most popular sport, with ∼265 million active players, 300,000 clubs, and 5 million referees worldwide (1). Despite this vast participation, elite success is highly concentrated, with only ∼124,000 players (i.e., 0.04%) classified as professionals by the Fédération Internationale de Football Association (FIFA) (1). The English Premier League is widely regarded as the strongest league globally (2), while the Ballon d'Or and The Best FIFA Men's Player award represent the highest individual honors in the sport (3).

Performance in soccer is determined by a combination of individual skills and the interaction and integration of these skills among players within the team. While technical and tactical abilities (e.g., ball control, passing, receiving, feints, scanning, positioning) are considered predominant, physical capacities such as sprinting speed, agility, repeated sprint ability and muscular power must also be highly developed for a player to succeed (4–6). Achieving this level of performance requires exceptional talent and many years of deliberate practice to fully realize a player's potential. Analyses of professional and major award-nominated players indicate an average peak performance age of 27–28 years, although there is considerable variation around this mean (3).

Information regarding the training and development of youth talents who successfully progress to professional soccer remains both limited and contradictory. For instance, the reported age of entry into soccer for both elite and sub-elite players ranges from 4 to 12 years (7–12). Although it is possible to become a successful player through participation in other sports during childhood and adolescence (7–9, 11, 12), it remains unclear whether such diversification is necessary to reach the highest levels of performance. Furthermore, research presents conflicting evidence regarding the progression of training volume during childhood and youth among players who eventually succeed at the senior level (7, 10–13). Helsen et al. (10) reported a gradual and linear increase in total training volume among Belgian upper league and national team players from the 1990s, more specifically 5–7 h per week at ages 6–8, 8–9 h per week at ages 10–12, and ∼10 h per week at ages 14–16. Ward et al. (7) observed a more nonlinear trend among academy players from Premier League clubs, more specifically a total volume of ∼13 h per week at ages 9–10, 19–20 h per week at ages 11–12, and 12–14 h per week at ages 13–18. It should be noted that the studies by Helsen et al. and Ward et al. are 20–30 years old and, therefore, may not reflect current practices. However, there seem to be a trend toward an increasing proportion of organized training and a corresponding decline in unorganized training with age (7, 10–13), although varying quantification methods complicate direct comparisons.

Several talent development models have been proposed to optimize sporting talent at the senior level, with three overarching features and their underlying sub-factors highlighted within this context. First, athlete characteristics include genetic factors, birth date (relative age effect), anthropometric and physiological predispositions, technical and tactical skills, mental abilities, motivational orientation, and personality traits. Second, the environment encompasses elements such as access to training facilities, family support, coaches, training peers, clubs, academies, and federations. Third, the practice of training involves aspects such as early vs. late specialization, training volume, intensity, frequency, and exposure to high-level practice and/or competition during adolescence (14–22). Talent pathways in elite sport are highly non-linear. Athletes differ widely in maturation, training history, psychological traits, support structures, coaching environments, and sociocultural conditions. Case studies can unravel these complexities, showing how and why certain developmental experiences lead to elite performance in a specific athlete. They also capture rare, atypical, or exceptional developmental trajectories in which quantitative studies tend to treat as outliers and often exclude. In their highly cited systematic review, Sarmento et al. (22) encouraged future research to investigate the best performers and adopt a longitudinal and multidimensional perspective. Therefore, based on a mixed-methods case study design, the aim of this study was to explore and describe the training and development process during childhood and youth for a multiple Ballon d'Or-nominated soccer player.

## 2 Methods

### 2.1 Study design

We performed an intrinsic case study to capture in-depth information on the development process of a world-class soccer player. This investigation adopts a pragmatic realist approach, aiming for a comprehensive and balanced exploration of the athlete's developmental journey by integrating both objective (i.e., training data) and subjective (i.e., interviews) perspectives. Ontologically, we acknowledge the existence of a reality that can be studied but emphasize that its understanding is shaped by the context and the perspectives of those involved. Although phenomena can exist independently, their significance and interpretation are shaped by social and experiential contexts. From an epistemological standpoint, we adopt a relational perspective in which knowledge emerges through the interaction between researcher and participants. Insights are not uncovered in isolation but arise through dialogue, reflection, and the co-construction of meaning within the research process. This approach enables the integration of objective historical training records with subjective interpretations, offering a more comprehensive understanding of the player's development in real-world contexts. This study design and approach has been utilized in several recent investigations related to best practice training recommendation and talent development (23–26).

### 2.2 Case description and informants

The primary case subject is a Norwegian male world-class soccer player whose developmental trajectory from childhood to elite performance offers a unique opportunity to examine the interplay among long-term training, environmental influences, and psychological factors. He started with organized team practice at the age of 5 and played for age-specific local teams for ten years (six seasons with one club, and four seasons with another club) until he was selected by a Norwegian upper league club at age 15, where he played for one season. In the same year, he made his senior international debut for the national team, becoming the youngest Norwegian player ever. At age 16, he transferred to a Spanish top-division club, where he primarily trained with the first team while competing in matches for the reserve team. Over the subsequent four years, he was loaned to several upper-league European clubs. At the time of data collection, the player was 27 years old, 178 cm tall, weighed 68 kg, and played as a midfielder for a Premier League club, where he had spent the previous four seasons. His professional honors include winning the Copa del Rey and the FA Community Shield, receiving Player of the Year and Player of the Month awards in the Eredivisie, La Liga, and Premier League, and earning two Ballon d'Or nominations.

The father of the soccer player was the primary informant. He was the head coach for the local and age-specific teams the player participated in from ages 5 to 15. His previous soccer-specific experience includes 16 years as a professional midfielder for two Norwegian upper-league clubs. After retirement as an active player, he served as assistant coach for a local senior club in the second highest division, academy coach for a Spanish top club, and head coach for two upper-league clubs during the four seasons preceding this study. His formal education includes the UEFA Pro License, the highest coaching certification available in Europe.

The case subject and informant (hereafter referred to as “the player” and “the father”) signed an institutionally approved informed consent document to participate in the research project. The Regional Committee for Medical and Health Research Ethics waived the requirement for ethical approval for this study. The study was conducted in accordance with institutional ethical requirements. Approval for data security and handling was obtained from the national center for research data (reference number 437719).

### 2.3 Data collection and refinement

A pragmatic four-step procedure was used to collect and refine comprehensive information on the development process of the player, similar to previous studies (23–26):

1. Historical training data, including training records, match appearances, plans, and developmental logs from the ages of 5–16 were gathered from personal archives and documented by the father. The training logs were based on recorded time in different activities. Specific soccer training was categorized as organized team practice, unorganized (and individual) practice with the father, and unorganized practice alone or with friends.
2. A first in-depth interview with the father was conducted by the first author to document and contextualize the historical training data, as well as to provide qualitative insights into training philosophy, decisions, and environmental contexts. Through a structured interview process, training content, and the planning and execution of specific training at the macro-, meso-, and micro-levels were documented, resulting in a preliminary overview of the player's training during the analyzed period. He also provided valuable insights into the overall training philosophy, player characteristics, and surroundings. This interview was conducted in person and lasted approximately two hours.
3. Three semi-structured follow-up interviews with the father were conducted after preliminary analyses to clarify, expand upon, or validate findings from step 1 or 2. These interviews lasted 45–75 min.
4. Lastly, the father and player were involved in an extensive review and negotiation process to refine and ensure that the findings reflected their perspectives on the training and development process as accurately as possible. Inconsistencies were addressed through follow-up communication with the father via phone or mail.

All interviews were audio-recorded and transcribed. Formal translation and back-translation from native language to English were performed by the first and last author, respectively.

### 2.4 Analysis

Numerical information (i.e., annual training volume across age stages and soccer practice categories) was systematized in Microsoft Excel (Microsoft Corporation, Redmond, WA, USA). For the qualitative data related to (a) training practices, (b) athlete characteristics, and (c) environmental influences, we employed a systematic six-step procedure informed by Braun and Clarke's reflexive thematic analysis framework (27). First, all authors engaged in familiarization with the interview material and initial discussions (step 1). Next, preliminary themes were generated through level 1 and level 2 coding (step 2), which were then organized into five themes for training features, five for athlete characteristics, and three for environmental factors (step 3). The refinement of these themes involved collaborative discussions among the authors and a structured negotiation process with participants to ensure both accuracy and interpretive resonance (step 4). In step 5, themes were contextualized within the broader literature on training science. Finally, all authors jointly contributed to the manuscript preparation, with results verified and approved by all informants (step 6).

## 3 Results

**Figure 1:**
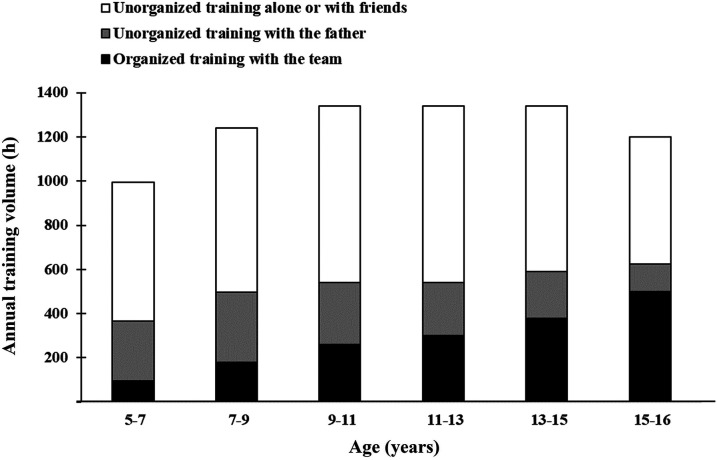
Annual training volume across age stages.

Figure 1 shows annual training volume across age stages. The total volume increased from ∼1,000 h·yr^−1^ at the age 5–6, reaching an apex of ∼1,300 h·yr^−1^ between the ages 9–15, followed by a slight decrease in the final analyzed training year. The player reached 5,000 and 10,000 h of accumulated training at ages 9.5 and 13.3, respectively. The proportion of organized training increased from ∼10 to ∼40%, while the proportion of unorganized training decreased from ∼90 to ∼60% throughout the analyzed period. The latter decrease was necessary to cope with the higher neuromuscular demands associated with organized training. Training alone or with friends accounted for 70%–80% of all unorganized training, while the remaining 20%–30% was performed with the father. Overall, large amounts of soccer-specific training were considered a necessity to become a world-class player (Table 1, Quote 1). Neither training for other sports nor physical conditioning was prioritized (Table 1, Quote 2 and 3), except for small and regular doses of speed/agility training (Table 1, Quote 4).

**Table 1:** Quotes made by the father about the player's training during childhood and youth.

Nr.	Quote
1	*“A huge number of youngsters worldwide are willing to go all in to become successful in soccer. It is therefore crucial to practice more and better than corresponding kids from Brazil, Argentina, Spain, England, etc. to develop superior technical/tactical skills necessary to handle the tempo at an international level.”*
2	*“X never participated in other organized sports. Because of the large doses of soccer practice, there was simply no room for other sports.”*
3	*“We did not prioritize physical conditioning during childhood and youth, except for speed and agility. X's physical capacity developed well through soccer practice, and his endurance on the pitch was superior compared to the other peers, most likely because of the large volume of soccer practice. Strength training was not implemented until the age 15 when he started to play for the local upper league senior club.”*
4	*“Prior to each organized soccer training session, 5–10 min with speed/agility cone drills were performed. Lack of speed and the ability to perform rapid changes of direction on the pitch were my main weaknesses during my own career, so I implemented small but regular doses of such training and hoped that X inherited his mother's genes instead of mine.”*
5	*“Training with older peers was crucial for X's development, even though he was born late in the year. In my opinion, this was the only solution to ensure sufficient training quality over time. We have to offer the kids adequate challenges according to their level of mastery. If they want to, and cope with the social aspects, I see no reason why talented kids should not practice with older peers.”*
6	*“During my own career, I struggled mentally when I made mistakes on the pitch. I was therefore concerned that X should keep trying, even though he failed, and I coached him accordingly. To me, the best learning from X's first upper league senior game was that he kept trying, even though he made 2–3 mistakes in the beginning of the match.”*
7	*“Ever since X was 5–6 years old, I was constantly focused on sufficient ball touches on every training session. Although I never mentioned this to him directly, this approach was always on my mind. I tried to maximize effective training time on each session by keeping instruction breaks to a minimum and avoid exercises where the players had to wait in line.”*
8	*“X's foremost qualities on the pitch as an adult include exceptional technical skills in terms of ball control, precise first and second touch, and high passing accuracy. His soccer intelligence is highly developed, and because of his oriented control and awareness, he manages to move the ball into free space and release himself from the pressure of the opponents. In addition, he possesses a high working capacity, creating space for his teammates and contributing significantly to the team's defensive play, counter-press and winning the ball far up in the opposition's’ half. This skillset was acquired through large amounts of targeted training drills and unorganized play during childhood and youth. During his mid-to-late teens and early 20s, he adapted and developed these skills to an even higher level by playing with better teammates and opponents.”*

Typical weekly training volume was 19–21 h·wk^−1^ at age 5–6, 25–28 h·wk^−1^ at 9–15, and 23–25 h·wk^−1^ at 15–16. Table 2 shows typical weekly training examples across age stages.

**Table 2:** Typical training week examples across age stages.

Age	Day	Unorganized training	Organized training
5–6 years	*Mon*	1:30–2:00 h (afternoon/evening)	1:00 h (evening)
	*Tue*	1:30–2:00 h (afternoon)	
	*Wed*	1:00–1:30 h (afternoon/evening)	1:00 h match (evening, only in season)
	*Thu*	1:30–2:00 h (afternoon)	
	*Fri*	1:00–1:30 h (afternoon/evening)	1:00 h (evening)
	*Sat*	4:00–5:00 h (midday)	
	*Sun*	4:00–5:00 h (midday)	
10–11 years	*Mon*	1:30–2:00 h (afternoon/evening)	1:30 h (evening)
	*Tue*	1:30–2:00 h (afternoon/evening)	1:30 h (evening)
	*Wed*	2:00–3:00 h (afternoon)	
	*Thu*	1:30–2:00 h (afternoon/evening)	2:00 h match (evening, only in season)
	*Fri*	1:30–2:00 h (afternoon/evening)	1:30 h (evening)
	*Sat*	5:00–7:00 h (midday)	
	*Sun*	5:00–7:00 h (midday)	
15–16 years	*Mon*	2:00 h (afternoon/evening)	1:30 h (midday)
	*Tue*	2:30 h (midday)	
	*Wed*	2:00 h (afternoon/evening)	2:00 h (midday)
	*Thu*	2:00 h (afternoon/evening)	2:00 h (midday)
	*Fri*	2:00 h (afternoon/evening)	1:30 h (midday)
	*Sat*	1:30 h (afternoon/evening)	1:30 h (midday)
	*Sun*		2:00 h match (evening, only in season)

There was no clear structure for when the unorganized training was performed alone, with friends or with the father. On weekdays, most unorganized training was performed right after school, either alone or with friends, but some of this training was also performed immediately after organized team practice in the evening. Unorganized training with the father was mainly performed in the weekday evenings (e.g., immediately prior to or after organized team practice) or in the weekends at midday.

The organized training sessions with the player's first club (i.e., age 5–10) were performed with 12–15 players and typically consisted of 10–15 min of dribbling/feints and ball control, 5 min of change-of-direction/speed drills, while small-sided play (6 vs. 6 or 7 vs. 7, pitch size ∼40 × 25 m) accounted for the remaining training time (45–75 min). Typical training sessions in the second club (i.e., 10–15 years) were performed with 20–25 players and consisted of 10–15 min passing/receiving drills, 5–10 min with change-of-direction/speed drills, 10–20 min ball control and possession drills, and finally 45–60 min with small-sided play (5 vs. 5 or 6 vs. 6, pitch size 15–25 × 25 m). The training sessions with the upper-league club (i.e., 15–16 years) consisted of both varying small-sided play, large-sided play, possession drills, formation drills and positioning drills.

The player trained and played consistently with 1–3-year older peers (Table 1, Quote 5). The number of games during childhood and adolescence was relatively restricted to allow more time for training. About 35 matches were played each season between the ages 5–9, and 35–40 matches per season between ages 10–15. In most season weeks, one game replaced one organized training session. The game format (number of players/pitch size/game duration) for the investigated player was 5 vs. 5/20 × 30 m/2 × 25 min when he was 5–6, 7 vs. 7/30 × 50 m/2 × 30 min when he was 7–9, and 11 vs. 11/60 × 100 m/2 × 35–45 min when he was 10–16 years.

The collective focus areas during small-sided training and games were playing out from the back, counter-pressing, and appropriate weight of passes. The player's individual focus areas during small-sided training and games were to seek and get the ball as often as possible, to challenge the opponents, and not being afraid of making mistakes (Table 1, Quote 6).

The father's training sessions (both organized and unorganized) followed a philosophy aiming at developing superior soccer-specific technical and tactical skills, enabling the player to cope with the tempo at the international level. The principle of specificity was paramount, and each session was performed with a high number of ball touches (Table 1, Quote 7). The basic skills were learned through isolated and targeted training drills, which also were embedded in what the father described as “joyful play”: informal, self-directed small-sided games with friends on the local turf that allowed the player to experiment, make mistakes and try again in an enjoyable setting. Across childhood and adolescence, the skills acquired in targeted drills were repeatedly reinforced and refined in these game-realistic situations and, ultimately, through match play. More difficult tasks were added as the player improved. The player was provided with individualized and appropriate challenges aligned with his level of mastery.

**Figure 2:**
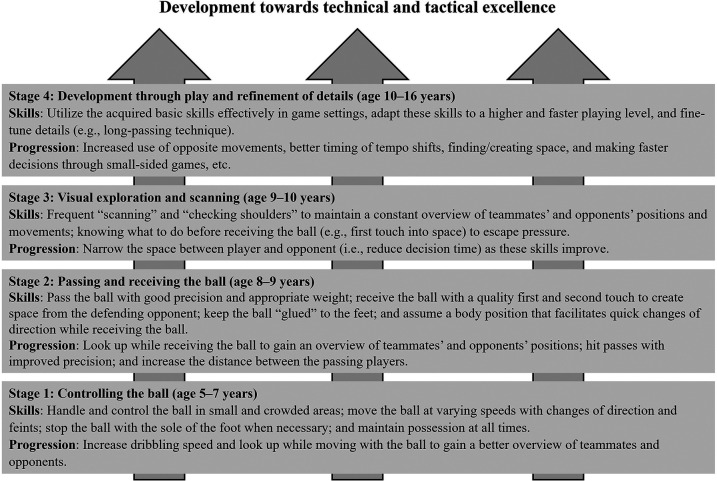
The main stages of the father's training philosophy.

The development of individual technical and tactical skills during childhood and adolescence followed four stages, which are described in Figure 2. The technical and tactical skills the player acquired at a young age also became prominent during adulthood (Table 1, Quote 8).

A selection of typical training drills from the player's childhood and adolescence can be viewed through the following link: https://www.youtube.com/playlist?list=PL0WXfI-ZvQ-oslxDVGLjH7LL7zmqJCEjp.

Table 3 describes key player characteristics underlying the successful talent development process, as highlighted by the father. These include exceptional motor talent, extreme passion for football and willingness to train, developmental orientation, confidence on the pitch, and a competitive mentality.

**Table 3:** Key athlete characteristics contributing to successful talent development.

Athlete characteristics	Descriptions
*Exceptional motor talent*	The investigated player exhibited a unique motor talent at a very young age. He could walk when he was nine months old, and he could ride a two-wheel bicycle already when he was 2 ½ years old. Moreover, he demonstrated an exceptional skiing technique on family tours, and combined with his aerobic endurance capacity, he could likely have been a well-performing cross-country skier. Regardless of the locomotion activities performed during early childhood, he learnt the basic techniques very fast.
*Extreme passion for football and willingness to train*	More important than natural sports talent was the player's inherent passion for football and motivation to practice. When he was only a few months old, he could not get enough of the ball in the playpen. When he became older, there was never a day without playing with the ball. Practically all recesses at school were devoted to soccer, and in the afternoon, he could play alone or together with friends on the local field for 1–3 h in addition to the organized training sessions. On Saturdays and Sundays, he could play unorganized soccer with different groups of kids for 4–7 h. Without this inner motivation and passion for football, he would never have been able to sustain the enormous amounts of practicing hours during childhood and youth.
*Developmental orientation*	Another key feature underpinning the player's success was his constant willingness to learn and search for development. At the age of 8–9 years, he evaluated his own games together with the father/coach in the car on the way home. The ability to reflect and identify strengths and weaknesses has constituted an important part of his overall development process. He also studied YouTube clips of world-class players, inspiring him to develop new technical skills, and parts of his technical repertoire as an adult player were learnt through such video analyses. He could also watch soccer games on television together with his father/coach and discuss tactical details. Although he gained considerable attention and praise for his soccer-specific skills as a youngster, he always sought further development.
*Confidence on the pitch*	The investigated player always trusted his soccer-specific skills, had faith in own abilities and exhibited confidence on the pitch. When he was selected for the first team of a Norwegian upper league club at the age of 15, he found his role as a central midfielder immediately, started directing the play and showed his teammates that he wanted to possess the ball. Interestingly, this confidence was not apparent in other areas of life, as he was generally a modest guy. For example, he did not dare to give a speech during his own confirmation dinner although only the closest family members were present.
*Competitive mentality*	According to the father/coach, the player exhibited a distinct winner instinct already from a very young age. He does everything to win a soccer game and hates to lose.

Table 4 describes key environmental factors underlying the successful talent development process. These include the father (and coach), access to training facilities all year round, and training peers.

**Table 4:** Key environmental factors contributing to successful talent development.

Environmental factors	Descriptions
*The father/coach*	As a former professional player in the two highest Norwegian divisions, the father possessed in-depth knowledge regarding the specific demands of soccer, more specifically what physical, technical and tactical skills should be emphasized and developed at what age stage. He played a particularly crucial role when the investigated player was 5–15 years old, as he was the head coach of the son's teams in this period. In addition, he supervised ∼20–30% of the son's self-organized sessions. The feedback and working tasks on these sessions formed the basis for the son's technical/tactical focus areas on the remaining soccer sessions. According to the father and coach, his most important contribution was developing the son's understanding of high training volume for attaining success. Overall, the father/coach spent 8–12 h per week together with his son on soccer training (both organized and unorganized) and games over a 10-year period. Furthermore, they spent numerous hours together in the car back and forth from games and practices, in addition to watching weekly soccer games on television. These served as discussion arenas where individual technical/tactical or team performances were commented on and evaluated. This process further developed and reinforced the son's understanding of play. The concurrent role as father and coach was never problematic for any of the parties. The player never received any extra benefits during regular team practice. In fact, the father/coach demanded more and was slightly stricter with his son than the other teammates during these sessions. For example, the son was never nominated as team captain during games if the father/coach was present, however, the assistant coaches nominated him a few times when the father (and head coach) was not present. Overall, the father-son relationship never abrased, and the son never bore any grudges against his father for being unfairly treated.
*Access to training facilities all year around*	Sufficient access to training facilities during childhood and youth has been paramount for the player's success. All sessions during the summer were performed on small- or moderate-sized soccer pitches in the neighborhood. Training during the winter was more challenging, as the local pitches were covered by snow for approximately three months per year. However, his father gained access to a school gymnasium nearby, providing opportunities for self-organized training alone or together with the father. Moreover, when the father became an assistant coach for one of the local senior elite clubs, he got access to an indoor soccer field. They arrived at the hall in good time before the adult's training, providing the investigated player opportunities to practice for 30–45 min. When the adults trained on the pitch, the investigated player played with the ball on the sideline or watched the session. After the adults were finished and went to the wardrobe for showering, the investigated player practiced in the hall for a further 30–60 min before he went home together with his father. In this way, the player attained large training volumes also during the winter.
*Training peers*	The training peers were important contributors to the investigated player's performance development. Because he was clearly more skillful than the local peers of the same age, he played upwards (i.e., with 1–3 years older teammates) on all organized training sessions and games, providing appropriate challenges. He also played with many of the same children on unorganized sessions in the afternoon, weekends, or at school. They matched very well, both socially and on the pitch. In this way, each session acted as youth play, not an obligation.

## 4 Discussion

This is the first study to offer in-depth and longitudinal scientific insight into the training and development process of a world-class soccer player. His training during childhood and adolescence was characterized by 1,000–1,300 annual hours of soccer training. Unorganized training accounted for most of the total, although this proportion gradually decreased from approximately 90% to 60% over the analyzed period, corresponding to an increase in organized training from 10% to 40%. The unorganized training with his father adhered to a clear philosophy where basic skills were learned through isolated and targeted drills in form of ball control, passing, receiving, feints, visual exploration and scanning. These skills were subsequently reinforced and refined in more game-realistic settings during unorganized play with friends on the local turf, and finally through match play. Key developmental factors underlying his successful progression included the player's multidimensional motor talent, an extreme passion for football, training with 1–3-year-older peers, a learning-oriented mindset, a supportive and knowledgeable father, and year-round access to good training facilities.

The large training volumes during childhood and adolescence represent the most noticeable finding in the present study. These are consistent with publicly available athlete biographies or non-scientific records of other exceptional talents across sports, for example Tiger Woods (golf), Simone Biles (gymnastics) and Michael Phelps (swimming). The ∼1,300 annual training hours performed by the player in the early teens are comparable to those of world-class senior endurance and tennis athletes (23–25). Moreover, the 5,000 and 10,000 h of accumulated practice at 9.5 and 13.3 years of age exceed previous reports of elite soccer players by a wide margin. For example, Premier League academy players, Belgian elite players (most of whom selected for the 1994 World Cup), and Norwegian professionals reached ∼1,200 and ∼4,600 h (7), ∼2,100 and ∼4,200 h (10), and ∼2,000 and ∼4,000 h (13) of accumulated training hours at the same ages, respectively. Although caution is warranted when comparing data across studies due to possible variations in quantification methods, these previous studies all included both organized and unorganized training. While exact volumes cannot be recommended due to the influence of other constraints, the amount of soccer-specific team practice during childhood is associated with higher levels of expertise (22). One may argue that the exceptional training volumes observed in this case study may prompt a paradigm shift in the development process of future talents. However, it is reasonable to assume that other world-class athletes have achieved success with considerably lower training volumes at the same age. The unusually high training volumes described here should not be interpreted as general recommendations for average youth players or non-elite settings.

The present player reduced the proportion of unorganized training as higher amounts of organized training were implemented, in line with previous studies (7, 10–13). This was done to cope with the higher neuromuscular demands associated with the organized training. Taken together, previous and present findings suggest that large doses of soccer practice can be tolerated prior to puberty, while organized training at an elite senior level requires longer recovery. For example, the annual training volume in a group of German upper-league and national team players (>22 years) was “only” 546 ± 125 h·y^−1^ (11) representing less than half the amount of the present player's total training volume in the early teens. Notably, most professional players engage in additional activities such as video analysis and psychological interventions that are not considered training *per se* but nevertheless require substantial time. As players become older, gain more muscle mass, and improve their fitness level, more and faster high-intensity actions are performed (28), in turn requiring longer recovery. The congested fixture schedules in the very best leagues constitute a major limitation for how much total training an adult player can handle.

Early specialization vs. diversified training has been the subject of considerable debate over the last decades (14–22). Although talent development studies have revealed considerable variation both within and across sports regarding routes to masterclass, there has been a general and growing trend in support of versatile training and late specialization during childhood. It has been argued that excessive amounts of specific training at an early stage increase the likelihood of overuse injuries, less enjoyment, psychological stress, and reduced participation in sport (14–21). However, several soccer-related studies have reported that most elite players have not participated in other sports during childhood and adolescence (8–13), consistent with the present observations. One may argue that soccer is a versatile activity *per se*, involving kicking, running, jumping, change-of direction movements in response to opponents and teammates, however, this does not imply that alternative activities are harmful or detrimental.

Another interesting aspect of the player's training was the unorganized training with his father, which followed a clear philosophy and consisted of isolated, targeted training drills to develop fundamental technical and tactical skills. Information related to the implementation of effective strategies that develop individual soccer-specific skills is scarce (22), but this study offers novel insights into a stepwise and stage-driven approach with practical examples. The constant focus on sufficient ball touches in every training session aligns with general motor learning development theories, as repetition is the “mother of learning” (29). Moreover, a well-established myth in motor skill development is that the basis for later success is built during a “golden age”, and that it is difficult to learn certain skills once the golden age has passed. Interestingly, no consensus has been established as to what precise age is the golden one, and no studies have so far been able to verify this construct (30) although several developmental mechanisms are frequently cited in discussions of age-related sensitivity (e.g., early myelination of motor pathways and synaptic pruning during childhood). However, the emphasis on technical and tactical elements rather than physical capacities in the early training years for the present player is in line with most talent development models (14–21).

Although large training volumes from early childhood contributed substantially to the player's later success, additional factors were fundamental for the positive development process. Training quality reflects the degree of excellence related to how the training sessions are executed to optimize adaptations and/or improve overall performance (31). Numerous features affect training quality, including the performer's mental/psychological skills and personality traits (23–26, 31). Previous studies have shown that successful athletes possess high levels of dedication, motivation, confidence, and a strong sense of ownership of the training process (15, 18), and these attributes are also characteristic of the present player. Indeed, his passion, willingness to train and learning-oriented mindset clearly contributed to the development of expertise. Moreover, although the father downplayed his own role during the interviews, it is the authors' opinion that he was likely the most important person contributing to the player's success. He understood the demands of the game, served as a mastermind of the training content, ensured access to training facilities all year around, coached the player, provided appropriate feedback and served as a discussion partner. It is well-known that parents (and coaches) can have a significant impact on the development of athlete performance (14–21) and the present case is not the only example of a sporting phenomenon guided or driven by a parent from a very young age (e.g., Serena and Venus Williams in tennis, Tiger Woods in golf). However, parental influence has been little studied in the context of soccer, and more studies are needed regarding how parents and coaches can support talented soccer players as they move across key transition points in their sport career (22).

According to his father, training with 1–3-year older peers was crucial to ensuring sufficient training quality and positive development over time. In settings where the performer is constantly and adequately challenged, new skills and strategies develop through co-adaptation to opponents (15). Situating this practice within the broader literature, this strategy bears resemblance to the principles of biobanding, which aim to reduce maturity-related biases and optimize challenge (32). Biobanding can support individualized development by allowing players to move up or down when appropriate, and differences in maturation may also influence psychosocial responses such as self-regulation (33). In the present case, however, the player moved upward because he demonstrated superior technical–tactical skills for his age, not because he was early-maturing. Although he coped well with the older peers, both socially and on the pitch, caution is warranted when deciding to play upwards. According to Kelly et al. (34), coaches and practitioners should consider technical, tactical, physical, psychological, and social characteristics before making such decisions. Overall, the present and previous findings (22) indicate that an optimal balance between organized and unorganized training, specifically tailored to maximize enjoyment, motivation and coping seems to promote talented players toward higher levels of expertise.

Several limitations of the present study should be acknowledged. While previous research on talent development often relies on either quantitative or qualitative approaches (22), this study aimed to integrate both methods. For the qualitative component, the study was evaluated using Tracy's eight key markers of quality (35): (a) worthy topic, (b) rich rigor, (c) sincerity, (d) credibility, (e) resonance, (f) significant contribution, (g) ethics, and (h) meaningful coherence. As noted in the introduction, the topic is deemed worthy as it addresses critical questions regarding the training and development of a world-class soccer player. Rigor, sincerity, and credibility were pursued through the use of established data collection methods that generated extensive qualitative and quantitative information from multiple perspectives; careful review and negotiation processes to verify accuracy and resonance; and transparent reporting of methodological procedures and limitations. The study is considered to make a valuable contribution to both scientific understanding and applied practice. Ethical considerations included formal research approval and the adoption of respectful practices to maintain a collaborative relationship between researchers and participants. Although the case subject and primary informant were anonymized, they were aware that their identity could potentially be inferred from the information presented and provided informed consent under these conditions. Meaningful coherence was achieved by aligning study aims, epistemology, conceptual frameworks, methodology, and presentation of findings with the original project design.

A recurring challenge in case studies of training practices is ensuring the validity and accuracy of retrospective data. In this study, the primary sources were training records and interviews provided by the father/coach, supplemented by the player's review and validation. Because the dataset is derived from father–son accounts, there is a probable risk of recall bias, selective reporting, and subjective interpretation. Consequently, the findings should be interpreted with caution, recognizing the limitations imposed by the narrow range of informants and the subjective elements embedded in the data.

Accurately quantifying training load in soccer remains challenging due to the sport's intermittent nature, recovery periods, and fluctuating intensity. Recorded training time was the only measure applied in this study; however, this does not capture the intensity dynamics. More detailed measures derived from wearable technology, motion analysis and ratings of perceived exertion would provide a more comprehensive understanding of the athlete's developmental trajectory. Unfortunately, such information was not available in the present case.

While case studies can provide rich, context-specific insights, their capacity for generalization is inherently limited. This investigation is based on situation-dependent, non-replicable observations that likely reflect idiosyncratic conditions, in this case the atypical interaction between an exceptional player and an exceptional coach. Consequently, the findings may have limited relevance for players of average ability or for different coaching environments, and there is a risk that readers may misinterpret this record of exceptionality as prescriptive training guidance. The effects of similarly voluminous early training on typical athletes and typical coaching contexts remain unknown, and coaches and parents should therefore refrain from directly replicating the training volumes reported here without careful consideration of individual and contextual constraints. Indeed, pathways to elite performance vary considerably, and other players may have undergone comparable training without achieving similar outcomes. Thus, the findings should be interpreted with caution, and causal inferences should not be drawn.

The professional background of the authors, including prior and current leadership roles within the Norwegian Olympic Federation and sports-oriented high schools, represents both a strength and a potential source of bias. Collectively, the research team possesses over three decades of experience working with elite athletes, including national-level soccer players, providing unique access and contextual insight to enhance data collection and interpretation. At the same time, this familiarity may have influenced the researchers' interpretations.

### 4.1 Conclusions

This case study offers novel information regarding the training and development process for a world-class male soccer player. The investigated world-class player in this case study performed 1,000–1,300 annual hours of soccer training. Unorganized training accounted for most of the total training volume, but its proportion gradually decreased as the amount of organized training increased. The training he did alone with his father followed a clear philosophy in which fundamental skills were developed through isolated and targeted drills. These abilities were then reinforced and refined in more game-realistic situations during unorganized play with 1–3-year older peers on the local turf, and ultimately through match play. Key features that contributed to a successful training and talent development process included the player's multidimensional motor talent, an extreme passion for football and willingness to train, a learning-oriented mindset, a supportive and knowledgeable father, and year-round access to quality training facilities. Overall, the reported findings may be useful for players, coaches, sport institutions, sport governing bodies, and sport scientists involved in talent development processes. However, this exceptional combination of a highly talented player and an expert coach is rare, and such voluminous training may not be appropriate or generalizable to average players with typical coaching support.

## Data Availability

The datasets presented in this article are not readily available because all data and materials support the published claims and comply with field standards. To protect the anonymity of the informants, the transcribed interviews cannot be made publicly available. Requests to access the datasets should be directed to thomas.haugen@kristiania.no.
